# Development and characterization of a novel injectable thyroid extracellular matrix hydrogel for enhanced thyroid tissue engineering applications

**DOI:** 10.3389/fbioe.2024.1481295

**Published:** 2024-11-27

**Authors:** Liang Zhang, Houlong Long, Peng Zhang, Bin Liu, Shuheng Li, Rong Sun, Tongmei Diao, Feng Li

**Affiliations:** ^1^ Department of Thyroid and Breast Surgery, Tengzhou Hospital Affiliated to Xuzhou Medical University, Tengzhou, China; ^2^ Department of Thyroid and Breast Surgery, Tengzhou City Center People’s Hospital, Zaozhuang, Shandong, China

**Keywords:** hypothyroidism, thyroid extracellular matrix, injectable hydrogel, cell viability, thyroid hormone secretion, thyroid tissue engineering

## Abstract

Hypothyroidism, a condition characterized by decreased synthesis and secretion of thyroid hormones, significantly impacts intellectual development and physical growth. Current treatments, including hormone replacement therapy and thyroid transplantation, have limitations due to issues like hormone dosage control and immune rejection. Tissue engineering presents a potential solution by combining cells and biomaterials to construct engineered thyroid tissue. This study focuses on the development and characterization of a novel 3D injectable hydrogel derived from thyroid extracellular matrix (TEM) for thyroid tissue engineering. TEM hydrogels were prepared through decellularization of rat thyroid tissue, followed by extensive physicochemical and mechanical property evaluations. The TEM hydrogels exhibited properties similar to natural thyroid tissue, including high biocompatibility and a complex 3D ultrastructure. Thyroid hormone-secreting cells cultured in TEM hydrogels demonstrated superior viability, hormone secretion, and thyroid-related gene expression compared to those in traditional type I collagen hydrogels. The study also confirmed the significant retention of key growth factors and ECM proteins within the TEM hydrogels. The results indicate that TEM hydrogels can provide a biomimetic microenvironment, promoting the long-term survival and function of thyroid cells, thus holding great promise for the treatment of hypothyroidism. This research contributes a potential new avenue for thyroid tissue engineering, offering a promising alternative for hypothyroidism treatment.

## 1 Introduction

Hypothyroidism is a condition characterized by reduced synthesis and secretion of thyroid hormones or insufficient physiological effects of these hormones, resulting in decreased metabolism. This disease can affect intellectual development and impair physical growth. Congenital factors, such as congenital hypothyroidism and autoimmune thyroiditis, as well as acquired factors, such as thyroidectomy or radioactive iodine therapy, can lead to the onset of hypothyroidism (Acosta et al., 2024; Vasilopoulou et al., 2024). Common treatments for hypothyroidism include hormone replacement therapy and thyroid transplantation. Oral hormone replacement therapy is a relatively simple and inexpensive alternative that usually requires lifelong treatment. However, due to the lack of a natural thyroid feedback mechanism for hormone secretion, hormone replacement therapy often encounters issues of over- or under-use of thyroid hormones (Razavi et al., 2024). On the other hand, the lack of donor organs, chronic immune rejection, and the necessity for long-term immunosuppression greatly limit the efficacy of thyroid transplantation (Petranović Ovčariček et al., 2024). Thus, tissue engineering technology could be a potential solution to this problem. By combining cells and biomaterials to construct engineered thyroid tissue, it provides a potential alternative treatment for hypothyroidism. The selection and preparation of biomaterials are fundamental to constructing engineered thyroid tissue. Injectable hydrogels, with their flexibility, porosity, adjustable shape, and durability, have been widely used in tissue engineering (Liu et al., 2020). Hydrogels prepared from synthetic polymers, such as polyacrylic acid, polymethacrylic acid, and polyacrylamide, have good mechanical properties but their inherent biological inertness limits their application in tissue engineering (Alven and Aderibigbe, 2020). Hydrogels prepared from natural polymers, such as polysaccharides and polypeptides, although biologically active, are limited by their inability to mimic the complexity of the natural extracellular microenvironment (Tuancharoensri et al., 2023).

In recent years, extracellular matrix (ECM) hydrogels, prepared by removing immunogenic cells with detergents and lysing, digesting, and gelatinizing the remaining natural tissue components, have garnered attention in the field of tissue engineering (Song et al., 2023). ECM hydrogels obtained through decellularization techniques have low immunogenicity, high biocompatibility, and complex 3D ultrastructures (Barajaa et al., 2024). Furthermore, ECM materials are rich in cytokines and growth factors that induce and regulate cell growth, proliferation, and differentiation, providing a favorable microenvironment for cell adhesion, proliferation, and differentiation (Keane et al., 2015). Growth factors play a crucial role in tissue repair and regeneration. For example, vascular endothelial growth factor (VEGF) promotes angiogenesis, improving tissue blood supply, thereby supporting cell proliferation and tissue regeneration (Ferrara, 2024). Transforming growth factor-beta (TGF-β) plays an important role in cell proliferation, differentiation, and ECM production (Massagué, 2012). Hepatocyte growth factor (HGF) promotes cell proliferation, migration, and survival through the c-Met receptor-mediated signaling pathway (Nakamura et al., 2011). Fibroblast growth factor (FGF) is widely involved in cell growth, development, and tissue repair (Volckaert and De Langhe, 2014). Epidermal growth factor (EGF) and platelet-derived growth factor (PDGF) also play key roles in cell proliferation, differentiation, and tissue repair (Kubota and Takigawa, 2011). The presence of these growth factors within the ECM hydrogel is essential for enhancing the viability and functionality of seeded cells, making them critical components in the context of tissue engineering. Additionally, ECM hydrogels can be delivered to damaged tissue sites *via* minimally invasive injection, offering advantages such as minimal invasiveness, safety, and high controllability, thus enhancing their clinical applications (Berry-Kilgour et al., 2021). Internationally, ECM hydrogels have been used in the repair and reconstruction of various tissues, including the heart, cartilage, skin, liver, tendons, dental pulp, and corneal stroma (Edgar et al., 2020). However, research on thyroid extracellular matrix (TEM) hydrogel materials is rare.

In this study, we report the formulation and characterization of a novel 3D injectable hydrogel material prepared from TEM. We seeded thyroid hormone-secreting cells into this hydrogel to construct engineered thyroid tissue. We first provided extensive characterization of the internal structure, mechanical properties, and physicochemical properties of the TEM hydrogel. The study showed that the TEM hydrogel exhibited elastic modulus, ECM composition, and growth factor reserves similar to those of the natural thyroid. Compared to traditional Collagen I hydrogels, thyroid hormone-secreting cells seeded in the TEM hydrogel demonstrated superior cell viability, thyroid hormone secretion function, and thyroid-related gene expression. The similarity in physicochemical and mechanical properties between the TEM hydrogel and the natural thyroid provides a more authentic microenvironment for the survival of thyroid hormone-secreting cells. Our results indicate that TEM hydrogel possesses excellent mechanical properties and biocompatibility. It can provide a biomimetic 3D microenvironment for the long-term survival and function of thyroid hormone-secreting cells, and engineered thyroid tissue based on TEM hydrogel holds great promise for the treatment of hypothyroidism.

## 2 Methods

### 2.1 Preparation of thyroid extracellular matrix

Healthy adult rats (weighing approximately 250–300 g, Wistar strain) were obtained from the Experimental Animal Center of Xuzhou Medical University. All animal experiments were conducted in accordance with the guidelines of the Animal Ethics Committee of Xuzhou Medical University and were approved accordingly (Approval number: [XZMUEAEC-2023012]). Sodium pentobarbital (40 mg/kg) was administered intraperitoneally for anesthesia to ensure the animals were pain-free during surgery. On a sterile operating table, scissors and forceps were carefully used to expose the neck area of the rat, identifying and isolating the thyroid tissue. Other tissues were covered with saline-moistened gauze to prevent drying and damage. The thyroid tissue was carefully separated from the surrounding tissues using dissection scissors and forceps, ensuring the integrity of the thyroid tissue. The obtained thyroid tissue was immediately placed in cold phosphate-buffered saline (PBS) to remove blood and fat tissue.

**FIGURE 1:**
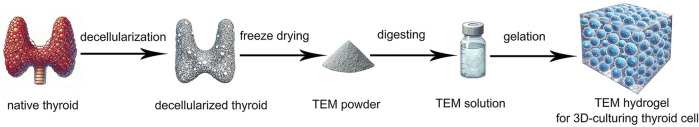
Schematic Diagram of the Preparation of Thyroid Extracellular Matrix (TEM) Hydrogel for 3D Culturing of Thyroid Cells. The process includes decellularization of native thyroid tissue, freeze-drying to obtain TEM powder, digestion to form TEM solution, and gelation to create TEM hydrogel for 3D cell culture.

The thyroid tissue blocks were placed in deionized water and shaken at 4°C for 24 h to remove extracellular soluble proteins and sugars. The tissue was then rinsed with deionized water three times, each for 5 min. The tissue blocks were placed in a 0.5% sodium dodecyl sulfate (SDS, Sigma-Aldrich, L3771) solution and shaken at room temperature for 24 h. After processing, the tissue was rinsed three times with deionized water, each for 5 min. The tissue blocks were then placed in a 1% Triton X-100 (Sigma-Aldrich, T8787) solution and shaken at room temperature for 24 h. After processing, the tissue was rinsed three times with deionized water, each for 5 min. The tissue blocks were then placed in deionized water and shaken at 4°C for 24 h. Finally, the tissue was rinsed three times with deionized water, each for 5 min, to obtain purified decellularized thyroid tissue (Figure 1).

### 2.2 Evaluation of decellularization efficiency

The efficiency of thyroid decellularization was systematically evaluated by measuring DNA, sulfated glycosaminoglycans (SGAG), and collagen content before and after decellularization. Thyroid tissues, 100 mg each, before and after decellularization were frozen and ground into powder and then dissolved in the appropriate extraction buffer. DNA was extracted and quantified using the PicoGreen DNA quantification kit (Invitrogen, P7589), and fluorescence intensity was measured using a fluorescence microplate reader (SpectraMax i3x, Molecular Devices) (excitation wavelength 480 nm, emission wavelength 520 nm). The DNA content before and after decellularization was compared to calculate the decellularization efficiency. SGAG content was measured by digesting the tissues with papain (Sigma-Aldrich, P4762) in a 60°C water bath for 24 h, followed by dimethylmethylene blue (DMMB) staining (Sigma-Aldrich). The content was calculated using the standard curve method and compared before and after decellularization. Collagen content was extracted and quantified using the Sircol collagen assay kit (Biocolor, S1000), with absorbance measured at 540 nm using a fluorescence microplate reader. The content was calculated using the standard curve method and compared before and after decellularization.

The efficiency of thyroid decellularization was further evaluated through hematoxylin and eosin (HE) staining, Masson’s trichrome staining, and scanning electron microscopy (SEM). Thyroid tissues, before and after decellularization, were cut into 5 µm thick sections, fixed in 4% paraformaldehyde (Sigma-Aldrich) for 15 min, and washed three times with PBS, each for 5 min, before HE staining. The stained sections were observed and imaged using an optical microscope (Leica DM 2000, Leica Microsystems) to evaluate tissue structure and cell nuclei removal. For Masson’s trichrome staining, tissue sections were similarly fixed in 4% paraformaldehyde for 15 min, washed three times with PBS, each for 5 min, and then stained with Masson’s trichrome (Sigma-Aldrich). The stained sections were observed and imaged using an optical microscope to evaluate collagen fiber distribution and retention. For SEM observation, thyroid tissues, before and after decellularization, were cut into approximately 1–2 mm³ pieces and fixed in 2.5% glutaraldehyde (Sigma-Aldrich) at 4°C for 2 h. The fixed tissues were washed three times with PBS, each for 10 min, and then post-fixed in 1% osmium tetroxide (OsO₄, Sigma-Aldrich) at room temperature for 1 h. The tissues were then washed with PBS and dehydrated through a graded ethanol series (30%, 50%, 70%, 90%, and 100%), each for 15 min. After dehydration, the samples were dried using a critical point dryer (Hitachi HCP-2). The dried samples were sputter-coated with gold or palladium to enhance conductivity. SEM (Hitachi, S-4800) was used to observe and image the samples at low accelerating voltage to evaluate the cell structure, ECM retention, and fiber network integrity before and after decellularization.

### 2.3 Retention of key proteins before and after decellularization

Immunofluorescence staining was used to assess the retention of key proteins, including collagen I (Abcam, ab270993), collagen IV (Abcam, ab6586), fibronectin (Abcam, ab268020) and laminin (Abcam, ab108536). Thyroid tissue sections were fixed in 4% paraformaldehyde (Sigma-Aldrich) for 15 min, washed three times with PBS, permeabilized with 0.1% Triton X-100 (Sigma-Aldrich) for 10 min, and blocked with 10% normal goat serum (Sigma-Aldrich) for 30 min. Primary antibodies were added and incubated overnight at 4°C, followed by washing three times with PBS. Fluorescent secondary antibodies (Alexa Fluor 488, Abcam, ab150077) were incubated at room temperature for 1 h, washed three times with PBS, and counterstained with DAPI (Thermo Fisher Scientific) for 1 min. Samples were observed under a fluorescence microscope (Leica, DM 2000), and staining intensity and distribution were compared to assess the effect of decellularization on key proteins.

Mass spectrometry analysis was also used to evaluate protein retention. Thyroid tissues, 100 mg each, before and after decellularization were frozen and ground into powder. The powder was dissolved in lysis buffer (Sigma-Aldrich) sonicated (Branson Ultrasonics) and centrifuged (Centrifuge 5417R, Eppendorf) to collect the supernatant for total protein extraction. Protein concentration was measured using the BCA Protein Assay Kit (Thermo Fisher Scientific, 23,225). Samples were incubated with 10 mM dithiothreitol (DTT, Sigma-Aldrich) at 37°C for 1 h and alkylated with 55 mM iodoacetamide (IAA, Sigma-Aldrich) in the dark for 30 min. Urea was diluted to 2 M, and trypsin (Promega, V5111, 1:50, w/w) digestion was performed overnight at 37°C. The reaction was stopped and acidified with 0.1% formic acid (Thermo Fisher Scientific). Peptides were analyzed by liquid chromatography-tandem mass spectrometry (LC-MS/MS) using a NanoLC system (Thermo Fisher Scientific) and a mass spectrometer (Q-Exactive HF, Thermo Fisher Scientific). Data-dependent acquisition (DDA) was used, selecting the top 20 most intense signals for fragmentation. Proteome Discoverer (Thermo Fisher Scientific) was used to analyze the mass spectrometry data, identifying and quantifying peptides and proteins. Protein profiles before and after decellularization were compared to assess the retention of key proteins.

### 2.4 Preparation of thyroid extracellular matrix (TEM) hydrogel

Decellularized thyroid tissue blocks were freeze-dried (Labconco, FreeZone 2.5) and ground into fine powder in liquid nitrogen. The TEM powder was dissolved in PBS containing 0.1% trypsin (Sigma-Aldrich) and 0.01% ethylenediaminetetraacetic acid (EDTA, Sigma-Aldrich) and digested at 37°C for 24 h. The digest was centrifuged, and the supernatant was collected to remove debris. The TEM solution was adjusted to pH 7.4 and dialyzed through a 3.5 kDa membrane at 4°C for 48 h, with the dialysis buffer changed every 4 h. The dialyzed TEM solution was freeze-dried to obtain purified TEM powder. The purified ECM powder was dissolved in sterile PBS to prepare 10 mg/mL and 20 mg/mL TEM solutions, stirred at 4°C overnight to ensure complete dissolution. The TEM solution was aliquoted into pre-chilled molds and gelled at 37°C for 30–60 min. The formed hydrogels were stored at 4°C and could be preserved in a small amount of PBS in sealed containers.

### 2.5 Optimization of TEM hydrogel concentrations for cell viability

FRTL-5 cells (ATCC) were used in this study to evaluate the biocompatibility and functionality of TEM hydrogels. FRTL-5 cells are a rat thyroid cell line commonly used in thyroid research due to their ability to perform typical thyroid cell functions, such as synthesizing and secreting thyroid hormones. Their ability to replicate thyroid cell behavior under *in vitro* conditions makes them an ideal model for studying thyroid tissue engineering applications. FRTL-5 cells were trypsinized, washed twice with PBS, counted, and resuspended to 1 × 10^6^ cells/mL. A 1 mL cell suspension was mixed with 1 mL 20 mg/mL type I collagen solution, 20 mg/mL TEM solution, 40 mg/mL TEM solution and 60 mg/mL TEM solution at a 1:1 volume ratio to prepare 10 mg/mL type I collagen solution, 10 mg/mL TEM solution, 20 mg/mL TEM solution and 30 mg/mL TEM solution. The mixture was distributed into 24-well plates, 500 μL per well. The hydrogels were placed in a 37°C, 5% CO_2_ incubator for 1 h, and then the medium was added to cover the hydrogels, with the medium changed every 2 days. After 7 days of culture, cells were washed three times with PBS, each for 5 min. Then, 10 μL CCK-8 reagent (Dojindo) and 90 μL serum-free medium were added to each well and incubated in the dark for 2 h. The supernatant was transferred to a 96-well plate, 100 μL per well. Absorbance at 450 nm was measured using a microplate reader (Thermo Fisher Scientific), with each sample repeated three times, and the average value was taken. A relative absorbance change graph of cell viability was plotted to evaluate the effect of different culture times and hydrogel concentrations on cell viability. This experimental design aims to evaluate the effect of different TEM hydrogel concentrations on FRTL-5 cell proliferation and viability, in order to identify the most suitable hydrogel concentration for supporting thyroid cell culture and function.

### 2.6 Observation of hydrogel microstructure

For SEM observation, hydrogel samples (10 mg/mL and 20 mg/mL TEM hydrogels, and 10 mg/mL type I collagen hydrogel) were cut into 1–2 mm³ pieces and fixed in 2.5% glutaraldehyde at 4°C for 2 h. After washing with PBS, the samples were post-fixed in 1% osmium tetroxide at room temperature for 1 h. The samples were dehydrated through an ethanol series and dried using a critical point dryer. Finally, the dried samples were sputter-coated with gold or palladium and imaged using SEM (Hitachi, S-4800) to examine the hydrogel microstructure.

### 2.7 Mechanical properties measurement

To compare the mechanical properties of different hydrogels, standard-sized samples (5 mm × 5 mm) were cut from fresh native thyroid tissue, 10 mg/mL type I collagen hydrogel, and 10 mg/mL and 20 mg/mL TEM hydrogels. All samples were hydrated in PBS for 1 h before testing. Hydrated samples were fixed in the grips of a mechanical analyzer (Zwick/Roell, BZ2.5/TN1S) to ensure stability and uniform force during testing. An initial preload of 0.015 N was applied to eliminate initial relaxation and stress concentration, and the initial length of the samples was recorded. A uniaxial tensile test was conducted at a speed of 1 mm/min with a preload of 0.003 N until the sample broke. The force-displacement curve was recorded to obtain the stress-strain curve. The elastic modulus (kPa) was calculated from the slope of the linear portion of the stress-strain curve. The toughness (N/m) was calculated from the area under the force-displacement curve.

### 2.8 Quantification of growth factors

Samples of native thyroid, 10 mg/mL type I collagen hydrogel, 10 mg/mL TEM hydrogel, and 20 mg/mL TEM hydrogel were cut into small pieces and placed in 500 µL of lysis buffer containing protease inhibitors (Complete Mini, Roche) for growth factor extraction. The samples were sonicated at 40% power for 30 s, with 30-second intervals, repeated 10 times to ensure thorough lysis. The lysates were centrifuged at 12,000 g for 10 min at 4°C, and the supernatant was collected for protein extraction. The final volume of the supernatant for each sample was adjusted to 500 µL with PBS. Specific ELISA kits were used to detect the content of growth factors, including VEGF (R&D Systems, RRV00), TGF-β (R&D Systems, RTB100B), HGF (R&D Systems, MHG00), FGF (R&D Systems, MFB00), EGF (R&D Systems, DY3214), and PDGF (R&D Systems, MBB00). Samples were diluted 1:10, and 100 µL was added to each ELISA plate well. The procedure followed the kit instructions, including sample incubation, washing, and substrate solution addition. The incubation was performed at room temperature for 1–2 h. After adding the chromogenic substrate (TMB, R&D Systems), the reaction was stopped, and absorbance was measured at 450 nm using a microplate reader. Growth factor concentrations were calculated from the standard curve prepared from each growth factor standard, ranging from 0–1,000 pg/mL. Each sample was tested in triplicate, and independent experiments were performed at least three times.

### 2.9 3D culture of thyroxine-secreting cells in hydrogels

FRTL-5 cells were trypsinized, washed with PBS, counted, and resuspended at 1 × 10⁶ cells/mL. A 1 mL cell suspension was mixed with an equal volume of 20 mg/mL type I collagen, 20 mg/mL TEM, and 40 mg/mL TEM to prepare 10 mg/mL type I collagen, 10 mg/mL TEM, and 20 mg/mL TEM solutions. The mixture was seeded into 24-well plates (500 μL per well) and incubated at 37°C, 5% CO₂ for 1 h. Medium was changed every 2 days.

After 3 and 7 days of culture, cells were washed with PBS and incubated with 10 μL CCK-8 reagent (Dojindo) and 90 μL serum-free medium for 2 h in the dark. The supernatant was transferred to a 96-well plate for absorbance measurement at 450 nm using a microplate reader (Thermo Fisher Scientific). Each sample was repeated in triplicate, and the average values were used to plot cell viability relative to culture time and hydrogel concentration.

After 3 and 7 days of culture, the culture medium supernatant was collected for tetraiodothyronine (T4) detection. The supernatant was centrifuged (1,000 rpm, 5 min) to remove cell debris. T4 concentration in the samples was measured according to the ELISA kit instructions (DRG International, EIA-1781) and compared with the control group.

After 3 and 7 days of culture, the hydrogels were dissolved with 0.1% collagenase (Sigma-Aldrich), and the cells were collected. Total RNA was extracted using TRIzol reagent (Invitrogen) and reverse-transcribed into cDNA using a reverse transcription kit (Takara). Thyroid-related gene thyroglobulin (TG) expression was detected by qRT-PCR. Data were analyzed using the ΔΔCt method, and gene expression was normalized to GAPDH. The gene primers for thyroglobulin (Tg) used are as follows: Upstream primer 5′-CAG​CTA​TGG​CAA​CAG​AAC​TT-3′, Downstream primer 5′-GTG​GCT​CCA​TTC​CCT​CAC​T-3'.

After 3 and 7 days of culture, immunofluorescence staining was performed. The hydrogel samples were embedded in optimal cutting temperature compound (OCT), frozen at −80°C, and sectioned. Sections were fixed in 4% paraformaldehyde (Sigma-Aldrich) for 10 min, treated with 0.1% Triton X-100 (Sigma-Aldrich) for 10 min, and blocked with 5% bovine serum albumin (BSA, Sigma-Aldrich) for 1 h. Primary antibodies against thyroglobulin (Abcam, ab156008) were incubated overnight at 4°C. Fluorescent secondary antibodies (Alexa Fluor 488, Abcam, ab150077) were incubated at room temperature for 1 h in the dark. Samples were counterstained with DAPI (1:1,000, Sigma-Aldrich) for 5 min in the dark. Samples were imaged using a confocal microscope (Nikon), and images were processed and analyzed using ImageJ software (NIH). The total fluorescence intensity per field was calculated, and the fluorescence intensity values for each sample were recorded.

### 2.10 Statistical analysis

Quantitative results are presented as means ± standard deviation (SD). The Shapiro-Wilk normality test was performed to verify that the data were normally distributed. Significant differences among groups were determined with the unpaired Student’s t-test or ANOVA followed by *post hoc* analysis for multiple group comparisons using SPSS 16.0 software (IBM). *p* < .05 was considered statistically significant.

## 3 Results

### 3.1 Evaluation of decellularization effectiveness

**FIGURE 2:**
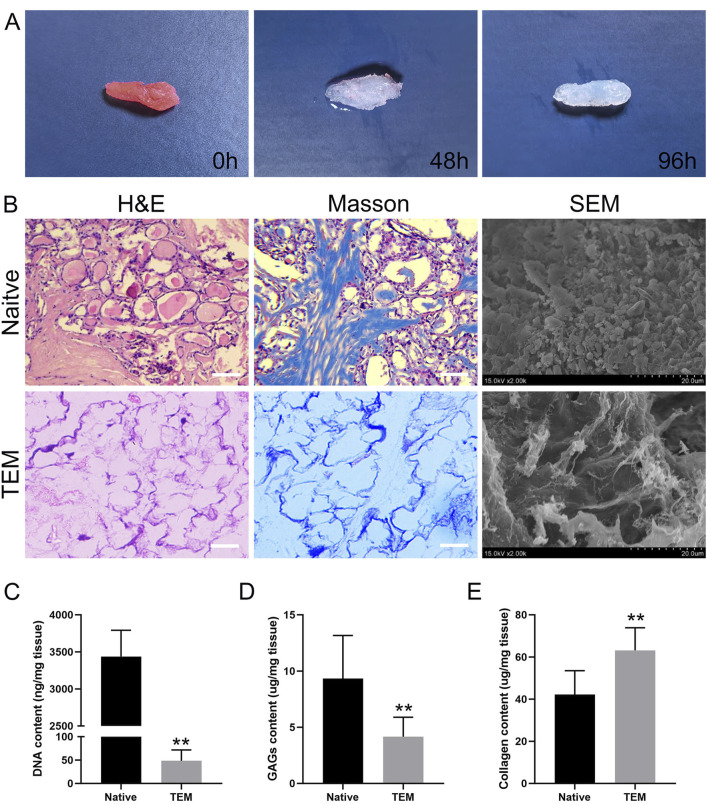
Evaluation of Decellularization of Thyroid Tissue and Characterization of Thyroid Extracellular Matrix (TEM). **(A)** Macroscopic images of thyroid tissue at 0 h, 48 h, and 96 h of decellularization. **(B)** H&E, Masson’s trichrome staining, and SEM images of native thyroid tissue and TEM. Scale bars = 100 μm. **(C–E)** Quantitative analysis of DNA content, glycosaminoglycans (GAGs), and collagen in native thyroid tissue and TEM. All data represent means ± SD. (n = 6; **p < .01 versus native thyroid tissue).

The decellularization process of the thyroid tissue is illustrated over a 96-hour period. Initially, at 0h, the thyroid tissue appears pink, indicating the presence of cellular components and an intact native structure. After 48 h, the tissue exhibits a noticeable change in color, becoming paler as the cellular materials begin to be removed. By 96 h, the tissue is almost entirely translucent, signifying that the decellularization process has been largely successful, leaving behind a scaffold primarily composed of extracellular matrix (Figure 2A). This visual progression effectively demonstrates the efficiency of the decellularization protocol used in the study.

The decellularization effectiveness was systematically evaluated by performing HE staining, Masson’s trichrome staining, and SEM, and by measuring collagen content, SGAG content and DNA content.

HE Staining: HE staining of thyroid tissue before decellularization showed an intact tissue structure with clear cell nuclei, indicating the tissue had not been treated (Figure 2B). Post-decellularization, the tissue slices showed nearly complete removal of cell nuclei while maintaining the structural integrity of the tissue. This indicates that the decellularization process effectively removed cellular nuclei but preserved the main tissue structure and extracellular matrix.

Masson’s Trichrome Staining: This staining method evaluated the distribution and retention of collagen fibers in thyroid tissue before and after decellularization. Before decellularization, tissue slices showed uniformly distributed collagen fibers and clear cell nuclei. Post-decellularization, the blue staining of collagen fibers remained evident, indicating that the process preserved collagen fibers and other ECM components. Despite the removal of cell nuclei, the basic structure of the tissue remained unchanged, with collagen fibers evenly distributed (Figure 2B).

SEM Observation: SEM images showed dense cells and ECM in thyroid tissue before decellularization, with clear visibility of cell surfaces and intercellular spaces, indicating complex structures. Post-decellularization, cells were removed, leaving behind a fibrous network of ECM. SEM images showed that the fibrous network maintained its original structure, despite cell removal, demonstrating good retention of the ECM’s microstructure (Figure 2B).

DNA Content Measurement: The DNA content in thyroid tissue before decellularization was 3,438 ± 354 ng/mg dry weight, while post-decellularization, it was significantly reduced to 49 ± 23 ng/mg dry weight (Figure 2C). The DNA content after decellularization was significantly lower than 50 ng/mg dry weight, indicating that the decellularization process effectively removed cellular components.

SGAG Content Measurement: The SGAG content in thyroid tissue before decellularization was 9.33 ± 3.83 μg/mg dry weight, whereas after decellularization, it was 4.17 ± 1.72 μg/mg dry weight (Figure 2D). Approximately 50% of the SGAG content was retained post-decellularization, indicating that the process effectively preserved the SGAG components.

Collagen Content Measurement: The collagen content in thyroid tissue before decellularization was 42.17 ± 11.32 μg/mg dry weight, and after decellularization, it increased to 63.17 ± 10.68 μg/mg dry weight (Figure 2E). This suggests that the decellularization process effectively preserved the collagen content.

Overall, the above results concluded that the decellularization process was effective. It efficiently removed cellular nuclei while preserving the primary collagen fibers and ECM structure, providing a solid foundation for subsequent applications.

### 3.2 Retention of extracellular matrix proteins

**FIGURE 3:**
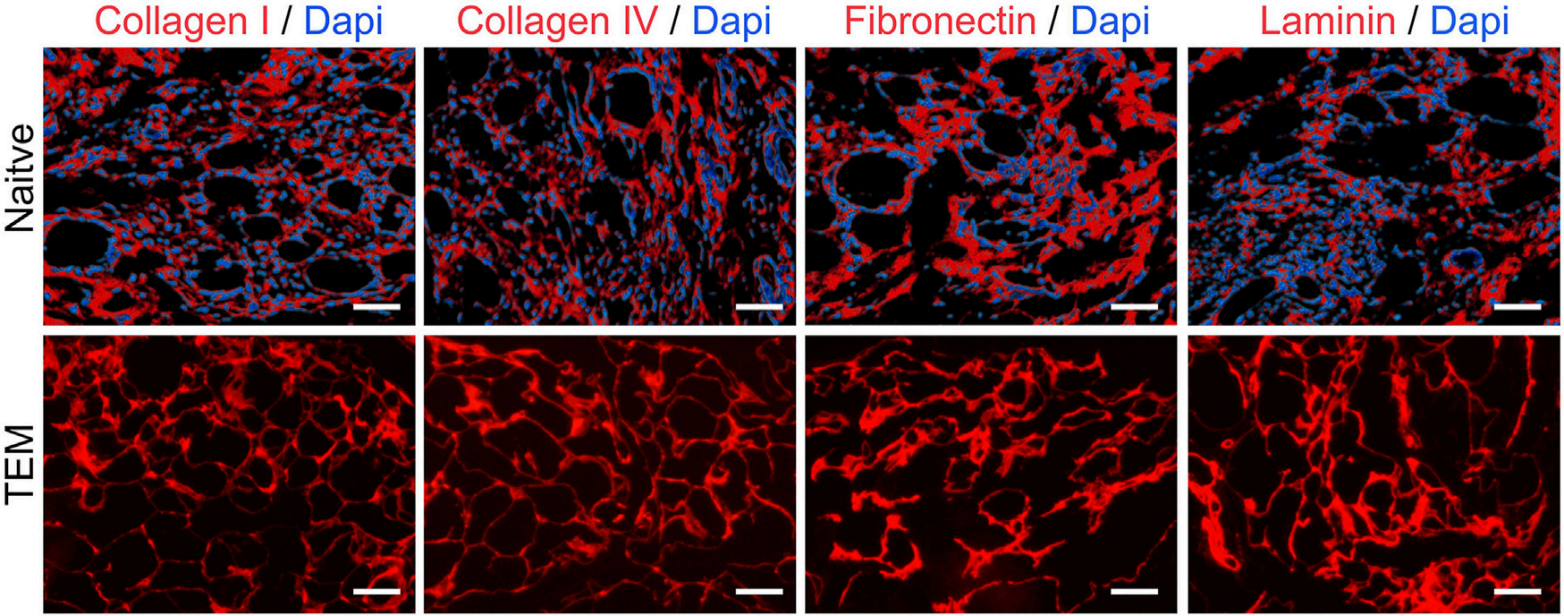
Immunohistochemical Analysis of ECM Proteins in Native Thyroid Tissue and Thyroid Extracellular Matrix (TEM). Collagen I, Collagen IV, Fibronectin, and Laminin are shown in red, with DAPI-stained nuclei in blue. Scale bars = 100 μm.

Immunohistochemistry Analysis: Immunohistochemistry was used to detect four major ECM proteins: collagen type I, collagen type IV, fibronectin, and laminin (Figure 3). Results showed that these proteins were widely present in the basement membrane and vascular structures post-decellularization, indicating effective retention of essential ECM proteins while removing cellular components.

Mass Spectrometry Analysis: Mass spectrometry revealed the retention of various protein types before and after decellularization (Table 1). Key structural and functional proteins were retained in the ECM post-decellularization, including most laminins and some collagens, fibronectins, elastins, proteoglycans, and nephron proteins. However, certain cytoskeletal proteins and adhesion molecules were undetectable after decellularization. These findings highlight the differential retention of protein types and provide essential insights into the impact of decellularization on thyroid tissue proteins.

**TABLE 1:** Mass spectrometry analysis of protein presence in native thyroid tissue and thyroid extracellular matrix (TEM).

Protein family	Gene	Description	Native thyroid	TEM
Collagens	COL5A3	Collagen alpha-3(V) chain	Y	N
Collagens	COL1A1	Collagen alpha-1 (1) chain	Y	Y
Collagens	COL11Al	Collagen alpha-1 (XI) chain	Y	N
Collagens	COL4A1	Collagen IV alpha-1 chain	Y	Y
Collagens	COL6A1	Collagen VI alpha-1 chain	Y	Y
Collagens	COL4A3	Collagen IV alpha-3 chain	Y	Y
Collagens	COL8Al	Collagen alpha-1(VIII) chain	Y	N
Collagens	COL7A1	Collagen alpha-1(VII) chain	Y	Y
Collagens	COL12Al	Collagen alpha-1 (XII) chain	Y	N
Collagens	COL4A2	Collagen IV alpha-2 chain	Y	Y
Collagens	COL6A3	Collagen alpha-3(VI) chain	Y	N
Collagens	COL14A1	Collagen alpha-1 (XIV) chain	Y	N
Collagens	COL5A2	Collagen alpha-2(V) chain	Y	Y
Collagens	COL15Al	Collagen alpha-1 (XV) chain	Y	N
Collagens	COL1A2	Collagen alpha-2 (1) chain	Y	Y
Collagens	COL3A1	Collagen alpha-1(III) chain	Y	Y
Collagens	COL18Al	Collagen XVIII alpha-1 chain	Y	N
Collagens	COL6A2	Collagen VI alpha-2 chain	Y	Y
Collagens	COL16Al	Collagen XVI alpha-1 chain	Y	N
Collagens	COL6A6	Collagen VI alpha-6 chain	Y	N
Laminins	LAMA2	Laminin subunit alpha-2	Y	Y
Laminins	LAMA5	Laminin subunit alpha-5	Y	Y
Laminins	LAMB2	Laminin subunit beta-2	Y	Y
Laminins	LAMB1	Laminin subunit beta-1	Y	Y
Laminins	LAMC3	Laminin subunit gamma-3	Y	Y
Laminins	LAMC1	Laminin subunit gamma-1	Y	Y
Laminins	LAMA4	Laminin subunit alpha-4	Y	Y
Laminins	LAMB3	Laminin subunit beta-3	Y	Y
Laminins	LAMC2	Laminin subunit gamma-2	Y	Y
Laminins	LAMAl	Laminin subunit alpha-1	Y	Y
Laminins	LAMB4	Laminin subunit beta-4	Y	Y
Fibronectins	FN1	Fibronectin 1	Y	Y
Fibronectins	FNDC1	Fibronectin domain-containing protein 1	Y	N
Elastins	ELN	Elastin	Y	Y
Proteoglycans	HSPG2	heparan sulfate proteoglycan core protein	Y	Y
Proteoglycans	AGRN	Agrin	Y	Y
Proteoglycans	BGN	Biglycan	Y	Y
Proteoglycans	DCN	Decorin	Y	Y
Proteoglycans	PRG4	Proteoglycan 4	Y	N
Nidogens	NID1	Nidogen-1	Y	Y
Nidogens	NID2	Nidogen-2	Y	Y
Cytoskeletal Proteins	TUBA1B	Tubulin alpha-1B chain	Y	N
Cytoskeletal Proteins	TUBB4B	Tubulin beta-4B chain	Y	Y
Cytoskeletal Proteins	VIM	Vimentin	Y	N
Cytoskeletal Proteins	DES	Desmin	Y	N
Adhesion Molecules	ICAM1	Intercellular adhesion molecule 1	Y	N
Adhesion Molecules	VCAM1	Vascular cell adhesion molecule 1	Y	N

The table lists proteins by family, gene, and description, indicating their presence (Y) or absence (N) in native thyroid tissue and TEM., protein families include collagens, laminins, fibronectins, elastins, proteoglycans, nidogens, cytoskeletal proteins, and adhesion molecules.

In summary, decellularized thyroid tissue effectively removed cellular components while retaining a substantial amount of ECM components. The experimental data validated the effectiveness and feasibility of the decellularization method used for preparing biological scaffolds.

### 3.3 Optimization of TEM hydrogel concentrations

**FIGURE 4:**
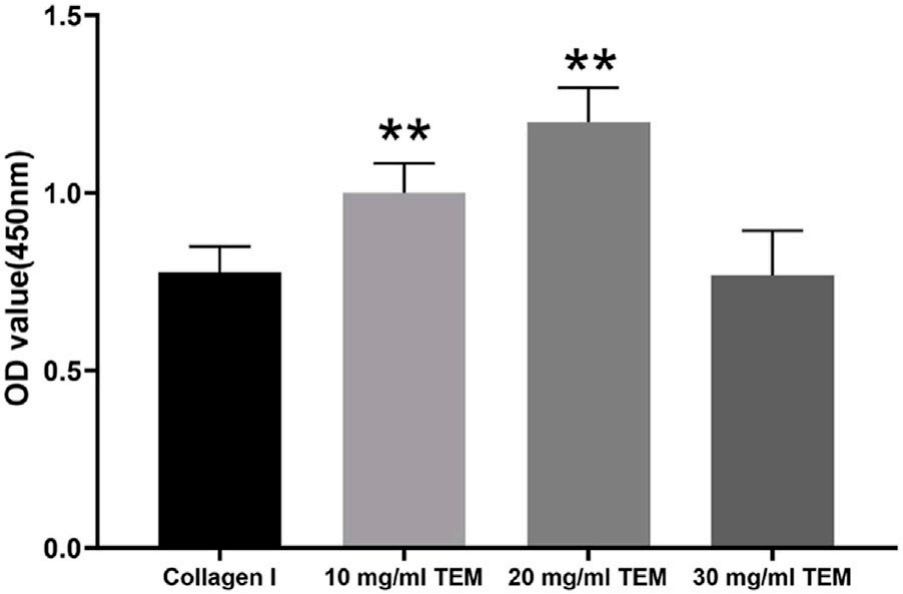
Cell viability of FRTL-5 cells cultured in 10 mg/mL type I collagen, 10 mg/mL TEM, 20 mg/mL TEM, and 30 mg/mL TEM hydrogels. Cell viability was assessed by measuring the optical density (OD) at 450 nm using the CCK-8 assay. All data represent means ± SD. (n = 6; **p < .01 vs. Collagen I).

In this study, we evaluated the effect of different concentrations of TEM hydrogels on the viability of FRTL-5 cells. The cell viability results obtained through the CCK-8 assay are as follows (Figure 4): The cell viability in the 10 mg/mL type I collagen group was 0.78 ± 0.07. As the control group, this showed relatively low cell viability, indicating limited support for FRTL-5 cells. The cell viability in the 10 mg/mL TEM hydrogel group was 1.00 ± 0.08, which showed a significant increase compared to the type I collagen group. The cell viability in the 20 mg/mL TEM hydrogel group reached 1.20 ± 0.10, the highest among all groups tested. However, when the hydrogel concentration was increased to 30 mg/mL, cell viability decreased to 0.77 ± 0.13, a level similar to the type I collagen group. This suggests that a high concentration of 30 mg/mL may have a negative impact on cell proliferation. Therefore, the 20 mg/mL TEM hydrogel provided an optimal environment for supporting thyroid hormone-secreting cells and maintaining a high proliferation rate in this study.

### 3.4 Observation of hydrogel microstructure

**FIGURE 5:**
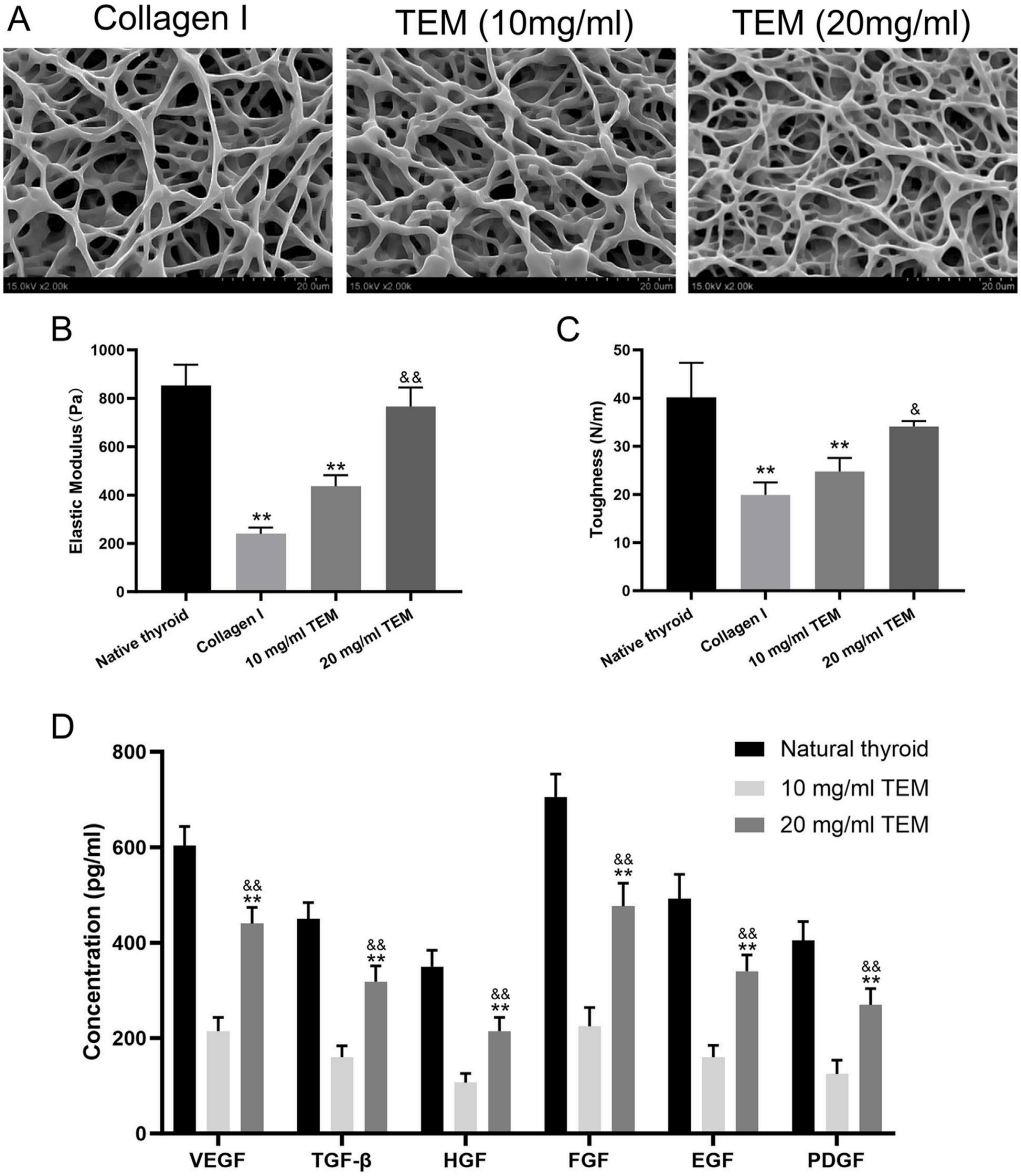
Structural, Mechanical, and Growth Factor Characterization of Thyroid Extracellular Matrix (TEM) Hydrogels. **(A)** SEM images of type I collagen hydrogel, 10 mg/mL TEM hydrogel, and 20 mg/mL TEM hydrogel. **(B)** Elastic modulus of native thyroid tissue, type I collagen hydrogel, 10 mg/mL TEM hydrogel, and 20 mg/mL TEM hydrogel. **(C)** Toughness of native thyroid tissue, type I collagen hydrogel, 10 mg/mL TEM hydrogel, and 20 mg/mL TEM hydrogel. **(D)** Concentrations of growth factors (VEGF, TGF-β, HGF, FGF, EGF, and PDGF) in native thyroid tissue, 10 mg/mL TEM hydrogel, and 20 mg/mL TEM hydrogel. All data represent means ± SD. (n = 6; **p < .01 vs. native thyroid, and *p* < .01 vs. 10 mg/mL TEM hydrogel).

The ultrastructure of 10 mg/mL type I collagen hydrogel, 10 mg/mL, and 20 mg/mL TEM hydrogels was observed using SEM. The 10 mg/mL type I collagen hydrogel exhibited a loose fibrous structure with irregular fiber arrangement and large pores. The ultrastructure of the 10 mg/mL TEM hydrogel was similar, showing irregular fiber arrangement and large pores. In contrast, the 20 mg/mL TEM hydrogel displayed a denser fibrous structure with smaller pores and more ordered fiber arrangement, which may better support cell adhesion and growth (Figure 5A).

### 3.5 Mechanical properties assessment

The mechanical properties of native thyroid tissue, 10 mg/mL type I collagen hydrogel, 10 mg/mL, and 20 mg/mL TEM hydrogels were tested. The elastic modulus, reflecting the material’s ability to resist deformation, was highest in native thyroid tissue (854 ± 85 Pa), significantly higher than all hydrogel groups. The 20 mg/mL TEM hydrogel (766 ± 79 Pa) followed, close to but slightly lower than native tissue. The 10 mg/mL TEM hydrogel (240 ± 26 Pa) and 10 mg/mL type I collagen hydrogel (436 ± 46 Pa) had significantly lower elastic moduli than native tissue (Figure 5B).

Toughness, reflecting the material’s ability to absorb energy and deform before breaking, was highest in native thyroid tissue (40.1 ± 7.2 N/m), significantly higher than all hydrogel groups. The 20 mg/mL TEM hydrogel (34.1 ± 1.1 N/m) followed, close to but slightly lower than native tissue. The 10 mg/mL TEM hydrogel (24.8 ± 2.8 N/m) and 10 mg/mL type I collagen hydrogel (19.9 ± 2.6 N/m) had significantly lower toughness than native tissue (Figure 5C).

In summary, mechanical testing shows that the 20 mg/mL TEM hydrogel is closer to native thyroid tissue and outperforms the 10 mg/mL TEM and type I collagen hydrogels, demonstrating potential as a tissue engineering material.

### 3.6 Growth factor content assessment

ELISA Analysis: ELISA was used to measure the content of key growth factors in native thyroid tissue, 10 mg/mL type I collagen hydrogel, 10 mg/mL, and 20 mg/mL TEM hydrogels. Native thyroid tissue had the highest growth factor content, indicating a rich reserve of growth factors supporting normal function and regeneration. The growth factor content in the 10 mg/mL TEM hydrogel was significantly lower than in the 20 mg/mL TEM hydrogel, indicating that the retention of growth factors in TEM hydrogels is closely related to their concentration. Specifically, the 10 mg/mL TEM hydrogel contained 215 ± 28 pg/mL of VEGF, 160 ± 24 pg/mL of TGF-β, 107 ± 19 pg/mL of HGF, 225 ± 39 pg/mL of FGF, 160 ± 25 pg/mL of EGF, and 125 ± 29 pg/mL of PDGF. In contrast, the 20 mg/mL TEM hydrogel contained 440 ± 34 pg/mL of VEGF, 318 ± 33 pg/mL of TGF-β, 215 ± 29 pg/mL of HGF, 477 ± 48 pg/mL of FGF, 340 ± 34 pg/mL of EGF, and 270 ± 34 pg/mL of PDGF (Figure 5D). This indicates that increasing the concentration of TEM hydrogel can significantly enhance the retention of growth factors, thereby increasing its bioactivity.

### 3.7 *In Vitro* 3D culture of thyroxine-secreting cells in hydrogels

**FIGURE 6:**
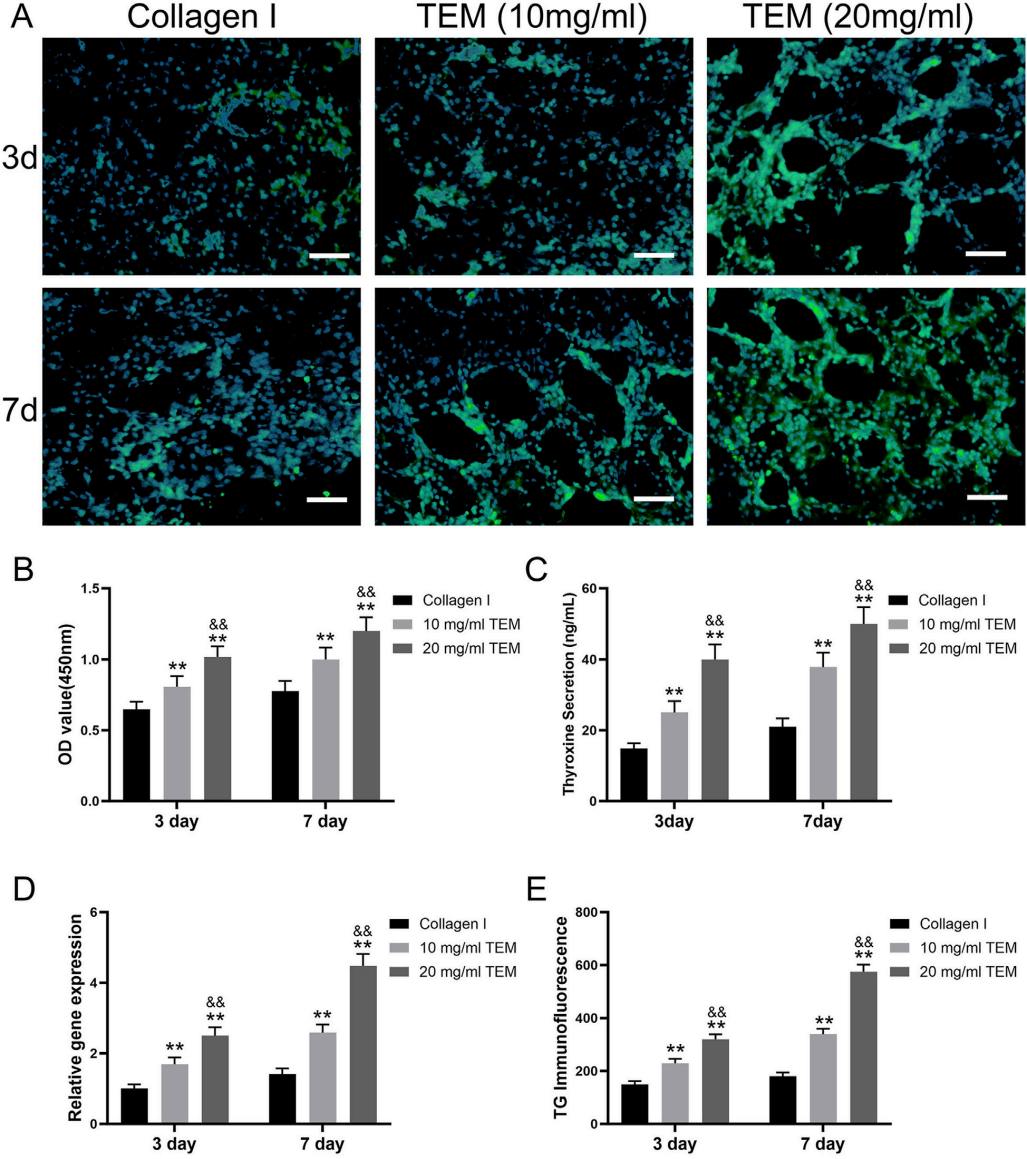
*In Vitro* Culture of Thyroxine-Secreting Cells in TEM Hydrogels. **(A)** Immunofluorescence images showing thyroglobulin (TG) expression (green) and nuclei (blue) in thyroxine-secreting cells cultured in hydrogel at 3 and 7 days. Scale bars = 100 μm. **(B)** Quantitative analysis of cell viability (OD value at 450 nm) at 3 days and 7 days. **(C)** Thyroxine secretion levels at 3 days and 7 days. **(D)** Relative gene expression of thyroglobulin (TG) at 3 days and 7 days. **(E)** Immunofluorescence intensity of thyroglobulin (TG) at 3 days and 7 days. All data represent means ± SD. (n = 6; **p < .01 vs. Collagen I, and *p* < .01 vs 10 mg/mL TEM).

Cell Viability Assessment: Cell viability in type I collagen hydrogel increased from 0.65 ± 0.06 at day 3 to 0.78 ± 0.07 at day 7. In the 10 mg/mL TEM hydrogel, cell viability increased from 0.80 ± 0.08 at day 3 to 1.00 ± 0.08 at day 7. In the 20 mg/mL TEM hydrogel, cell viability increased from 1.00 ± 0.08 at day 3 to 1.20 ± 0.10 at day 7 (Figure 6B). These results indicate that cell viability increased over time in all hydrogel groups, with higher ECM concentrations showing more significant increases, demonstrating the positive role of ECM in cell proliferation.

Tetraiodothyronine (T4) Secretion Measurement: T4 secretion in type I collagen hydrogel increased from 14.8 ± 1.5 ng/mL at day 3–21.0 ± 2.4 ng/mL at day 7. In the 10 mg/mL TEM hydrogel, T4 secretion increased from 25.0 ± 3.2 ng/mL at day 3–37.8 ± 4.0 ng/mL at day 7. In the 20 mg/mL TEM hydrogel, T4 secretion increased from 40.0 ± 4.2 ng/mL at day 3–50.0 ± 4.7 ng/mL at day 7 (Figure 6C). T4 secretion increased over time in all groups, with significantly higher levels in the high-concentration ECM hydrogel group, indicating that ECM concentration markedly promotes thyroid cell function.

TG Gene Expression Analysis: TG gene expression in type I collagen hydrogel increased from 1.00 ± 0.12 at day 3 to 1.41 ± 0.17 at day 7. In the 10 mg/mL TEM hydrogel, TG gene expression increased from 1.69 ± 0.19 at day 3 to 2.58 ± 0.23 at day 7. In the 20 mg/mL TEM hydrogel, TG gene expression increased from 2.50 ± 0.24 at day 3–4.48 ± 0.33 at day 7 (Figure 6D). All groups showed significant increases in TG gene expression over time, especially in the high-concentration ECM hydrogel group, indicating that ECM not only maintains cell viability but also enhances gene expression levels.

TG Immunofluorescence Staining: TG fluorescence intensity in type I collagen hydrogel increased from 150 ± 12 at day 3–180 ± 14 at day 7. In the 10 mg/mL TEM hydrogel, TG fluorescence intensity increased from 229 ± 17 at day 3–339 ± 20 at day 7. In the 20 mg/mL TEM hydrogel, TG fluorescence intensity increased from 319 ± 19 at day 3–575 ± 27 at day 7 (Figure 6E). Fluorescence intensity increased significantly over time in all groups, particularly in the high-concentration ECM hydrogel group, indicating a marked enhancement in protein expression.

In the 20 mg/mL TEM hydrogel, thyroid cells exhibited higher cell viability, T4 secretion, TG gene expression, and TG protein expression. Additionally, they formed more follicle-like cavity structures (Figure 6A). These cavities have central hollows, resembling the functional units of the thyroid *in vivo*, indicating that high-concentration TEM hydrogels can better mimic the natural microenvironment of thyroid tissue, thereby promoting the functionalization and organized growth of thyroid cells. This further demonstrates the potential application value of high-concentration TEM hydrogels in thyroid regenerative medicine.

## 4 Discussion

In this study, we evaluated the decellularization efficacy of thyroid tissue through a series of physicochemical analyses and morphological observations. The results demonstrated that the decellularization process effectively removed cellular components while preserving the main components of the ECM. These findings strongly support the potential of TEM in tissue engineering applications. Firstly, DNA content analysis showed a significant reduction in DNA content after decellularization, indicating effective removal of cellular components. The DNA content after decellularization was below 50 ng/mg dry weight, meeting the safety standards for residual DNA content in biomaterials (Zhang et al., 2021). Studies have shown that excessive residual DNA can trigger immune and inflammatory responses, thereby affecting the biocompatibility of the material (Zheng et al., 2018). Therefore, the significant reduction in DNA content after decellularization suggests that the method effectively reduces potential immunogenicity, providing a good premise for subsequent cell transplantation and tissue engineering applications. Secondly, glycosaminoglycan (GAG) content analysis showed that approximately 50% of the GAG content was retained after decellularization. GAGs are crucial components of the ECM, involved in biological processes such as cell signaling, adhesion, and proliferation (Campos et al., 2019). The retention of GAG content is essential for maintaining the biological functions of the ECM. Despite some loss of GAGs during decellularization, more than half of the GAGs were retained, indicating that the decellularization process somewhat protects the functional components of the ECM. Studies suggest that the presence of GAGs helps promote cell attachment and proliferation, thus supporting tissue repair and regeneration (Hamilton et al., 2022). Collagen content analysis showed a slight increase in collagen content after decellularization. Collagen is a major structural protein in the ECM, playing important roles in mechanical support and cell adhesion (Sorushanova et al., 2019). The increase in collagen content after decellularization could be due to the relative increase in collagen fibers after the removal of cellular components. This result suggests that the decellularization process effectively preserves collagen, contributing to the material’s mechanical performance and biocompatibility. Studies have shown that collagen is widely used in tissue engineering for promoting cell attachment, proliferation, and differentiation (Chen et al., 2019).

HE (hematoxylin and eosin) staining and Masson staining further verified the effectiveness of the decellularization process. HE staining showed almost complete removal of cell nuclei after decellularization, while the overall tissue structure remained intact. Masson staining showed that collagen fibers were retained and evenly distributed after decellularization. These staining results are consistent with the collagen content analysis, indicating that the decellularization process effectively removes cellular components while preserving the main structures and components of the ECM. This is crucial for constructing tissue engineering scaffold materials with good biocompatibility and mechanical properties (Nii and Katayama, 2021). Scanning electron microscopy (SEM) observations showed that native thyroid tissue displayed a dense network of cells and ECM with a complex structure. After decellularization, the samples showed a remaining fiber network of the ECM, indicating effective removal of cells and preservation of the ECM structure. SEM images showed that the fibrous network was retained after decellularization, further demonstrating the preservation of ECM microstructure. Studies suggest that the fiber network structure of the ECM is important for supporting cell adhesion and proliferation (Brown and Badylak, 2014).

In this study, thyroid tissue was processed using decellularization techniques to prepare a biological scaffold that retains key ECM proteins. The experimental results showed that the decellularized TEM exhibited good structural integrity. Multiple collagen types (including types I, III, IV, V, VI, and XI) were still detectable after decellularization, indicating that the process preserved most collagen proteins, ensuring the basic structure of the scaffold (Ikegami and Ijima, 2021; Song et al., 2018). Notably, collagen alpha-1(I) chain (COL1A1) and collagen IV alpha-1 chain (COL4A1) were still present after decellularization. These proteins are essential for maintaining the integrity of the basement membrane structure (Karamanos et al., 2021). Additionally, fibronectin (FN1) and laminin proteins (such as LAMC1, LAMA1, and LAMB1) were also detectable after decellularization, which contribute to cell attachment and functional maintenance of the scaffold (Rocha et al., 2017; Graf et al., 2021). Elastin (ELN) and proteoglycans (such as HSPG2, AGRN, and DCN) were also detected post-decellularization, indicating that the retention of these components helps maintain the elasticity and structural integrity of the scaffold (Kim et al., 2011). However, cytoskeletal proteins (such as TUBA1B and VIM) and adhesion molecules (such as ICAM1 and VCAM1) were not detected after decellularization, suggesting changes in cellular structural support and cell-cell adhesion. The absence of these proteins may affect the mechanical properties and biological functions of the scaffold (Chastney et al., 2024).

This selective retention phenomenon can be attributed to several mechanisms: first, ECM proteins such as collagen and laminin are highly cross-linked within the tissue, providing strong structural stability that can resist the physical and chemical disruption during the decellularization process (Ricard-Blum, 2011). Second, certain ECM proteins, such as collagen and laminin, are primarily located in the basement membrane and vascular structures, and are less associated with cell membranes, making them less likely to be removed during the decellularization process (Iozzo and Schaefer, 2015). Finally, decellularization reagents may selectively act on different types of proteins, effectively removing cellular components while having minimal impact on ECM proteins (Badylak, 2007). Retaining these key ECM proteins is significant for the functionality of tissue engineering scaffolds. ECM proteins not only provide physical support but also interact with cell surface receptors to regulate cell adhesion, migration, proliferation, and differentiation (Brown et al., 2012; Gu et al., 2019). This was confirmed in subsequent cell experiments. Specifically, FRTL-5 rat thyroid cells showed significantly higher viability, thyroid hormone secretion, thyroid-related gene expression, and immunofluorescence staining in high-concentration ECM hydrogels compared to low-concentration and type I collagen hydrogel groups.

We evaluated the effects of 10, 20, and 30 mg/mL TEM hydrogels on the proliferation and viability of thyroid hormone-secreting cells. Through CCK-8 assays, we found that the 20 mg/mL TEM hydrogel effectively promoted cell proliferation and viability, while increasing the concentration to 30 mg/mL resulted in a decrease in both proliferation and viability. This phenomenon can be primarily attributed to limitations in nutrient and waste diffusion. As the hydrogel concentration increases, the fiber network becomes denser, and the internal pore structure contracts. These structural changes directly affect the diffusion of oxygen, essential nutrients, and metabolic waste within the hydrogel, making it difficult for cells to obtain sufficient nutrients, while metabolic waste cannot be efficiently removed. Over time, cells in such a microenvironment experience reduced proliferation. Therefore, we believe that the 20 mg/mL TEM hydrogel provides a more balanced microenvironment for thyroid hormone-secreting cells, striking an optimal balance between nutrient and waste diffusion.

In this study, the mechanical properties of natural thyroid tissue, 10 mg/mL type I collagen hydrogel, and 10 mg/mL and 20 mg/mL TEM hydrogels were analyzed through mechanical testing. The elastic modulus is an important indicator of a material’s resistance to deformation. Natural thyroid tissue showed the highest rigidity and resistance to deformation. The elastic modulus of the 20 mg/mL TEM hydrogel was close to that of natural thyroid tissue, indicating its high potential for structural support. A higher elastic modulus helps maintain cell morphology and function, which is crucial in tissue engineering applications. Studies have reported that cells are sensitive to the rigidity of the matrix, and higher matrix rigidity helps maintain cell proliferation and differentiation (Matsuzaki et al., 2016; Siroha et al., 2022). Therefore, the 20 mg/mL TEM hydrogel, with its higher elastic modulus, may be more suitable for thyroid tissue engineering applications. Toughness reflects the ability of a material to absorb energy and deform before breaking, which is a composite indicator of material strength and ductility. In this study, the toughness of natural thyroid tissue was significantly higher than that of all hydrogel groups. The toughness of the 20 mg/mL TEM hydrogel was the next highest, close to but slightly lower than that of natural thyroid tissue. The toughness of the 10 mg/mL TEM hydrogel and 10 mg/mL type I collagen hydrogel was significantly lower than that of natural tissue. Higher toughness helps the material maintain integrity under dynamic stress, providing a longer-lasting and stable environment for cell growth. The high fiber density and tight structure of high-concentration ECM hydrogels can more effectively dissipate stress and absorb more energy, thereby improving their toughness. This property is crucial for withstanding dynamic stresses *in vivo*, as tissue engineering materials need to endure various mechanical stresses under physiological conditions (Li et al., 2022; Liu and Zhao, 2022).

In this study, the contents of several key growth factors in natural thyroid tissue, 10 mg/mL type I collagen hydrogel, and 10 mg/mL and 20 mg/mL TEM hydrogels were measured by enzyme-linked immunosorbent assay (ELISA). The results showed that the growth factor content in natural thyroid tissue was the highest, which is expected because natural tissues have a rich reserve of growth factors to support their normal functions and regenerative capacity (Martino et al., 2014). Notably, the growth factor content in the 10 mg/mL TEM hydrogel was significantly lower than in the 20 mg/mL TEM hydrogel, indicating that the retention of growth factors in ECM hydrogels is closely related to their concentration. This phenomenon may be attributed to the microstructural characteristics of ECM hydrogels. High-concentration ECM hydrogels form a denser three-dimensional network structure, which helps capture and retain growth factors (Diller and Tabor, 2022). Although decellularization leads to the loss of some GAGs, a significant proportion remains. Additionally, proteomic analysis (as shown in Table 1) reveals that certain proteoglycans, such as HSPG2, Agrin, Biglycan, and Decorin, are still retained. These residual GAGs and PGs can still bind to growth factors within the hydrogels, forming stable complexes through charge and steric interactions, thereby extending the retention time of growth factors in the hydrogels (Brizzi et al., 2012; Sodhi and Panitch, 2020).

In this study, the retention of key growth factors such as VEGF, TGF-β, HGF, FGF, EGF, and PDGF within the TEM hydrogel played a significant role in enhancing cell viability and function. These growth factors collectively contributed to the promotion of thyroid cell proliferation, differentiation, and tissue repair by providing crucial biochemical signals. The presence of VEGF facilitated improved nutrient supply and waste removal through enhanced vascularization, while TGF-β, FGF, and HGF supported cellular growth and tissue development through complex signaling pathways (Ciprian, 2015). Additionally, EGF and PDGF further augmented cellular activities by influencing cell proliferation and matrix synthesis (Cecerska-Heryć et al., 2022). The retention of these factors in the high-concentration TEM hydrogel highlights its bioactivity and ability to mimic the natural tissue microenvironment, which is crucial for successful tissue engineering applications.

The TEM hydrogel developed in this study showed significant ability to promote thyroid cell viability and function in a 3D *in vitro* culture system. The experimental results indicated that the 20 mg/mL TEM hydrogel outperformed the 10 mg/mL TEM hydrogel and the type I collagen hydrogel in multiple indicators, demonstrating a stronger promotive effect of high-concentration ECM on thyroid cells. High-concentration ECM hydrogels provide a richer and more complex ECM component, which plays an important role in cell adhesion, proliferation, differentiation, and function maintenance (Cain et al., 2008). For example, collagen types I and IV are crucial components of the ECM, providing structural support and interacting with cell surface integrin receptors to activate downstream signaling pathways that regulate cell proliferation and differentiation (Schiller et al., 2013). Additionally, GAGs in the ECM, such as hyaluronic acid and chondroitin sulfate, not only play roles in maintaining the structural integrity of the ECM but also participate in cell signal transduction and gene expression regulation (Lam et al., 2014). These GAGs interact with cell surface receptors to regulate cell proliferation, migration, and differentiation, thereby enhancing cell function. Growth factors in high-concentration ECM hydrogels, such as basic fibroblast growth factor (bFGF) and insulin-like growth factor (IGF), also play important roles in promoting cell proliferation and differentiation (Son et al., 2015). These growth factors bind to cell surface receptors and activate downstream signaling pathways (such as PI3K/Akt and MAPK/ERK pathways), promoting cell proliferation and differentiation and enhancing functional expression (Haarmann-Stemmann et al., 2009). Moreover, high-concentration ECM provides a better physical support environment, promoting cell-ECM interactions, thereby enhancing functional expression (Marklein and Burdick, 2010). This indicates that the bioactive components in the ECM play a key role in maintaining and enhancing thyroid cell function.

In the 20 mg/mL TEM hydrogel, thyroid cells formed more follicle-like cavity structures. The formation of cavity structures is mainly due to the ECM providing a three-dimensional scaffold that supports cell adhesion and growth and provides a microenvironment similar to *in vivo* conditions, which aids in spatial arrangement and organization of cells (Mangano et al., 2011). In high-concentration ECM hydrogels, the combined action of abundant bioactive molecules and suitable mechanical strength promotes the aggregation and functional structure formation of thyroid cells. Components of the ECM, such as collagen, fibronectin, and laminin, provide critical biological signals that interact with cell surface receptors to regulate cell behavior and tissue structure (Yue, 2014). In the 20 mg/mL TEM hydrogel, the high concentration of these components likely provided stronger growth and differentiation signals to thyroid cells, promoting the formation of cavity structures. The formation of cavity structures not only indicates enhanced survival and proliferation of cells in high-concentration ECM hydrogels but also suggests functional organization of cells. Thyroid follicles are the basic units of thyroid function, essential for thyroid hormone synthesis and secretion (Liang et al., 2022). The ability to form follicle-like cavity structures *in vitro* suggests that these cells in high-concentration ECM hydrogels grow in an environment very similar to the *in vivo* environment, which is significant for thyroid regeneration research.

## 5 Conclusion

In summary, 20 mg/mL TEM hydrogel significantly promotes thyroid cell viability, thyroxine secretion, TG gene expression, and protein expression by providing abundant extracellular matrix components and bioactive molecules. These results indicate that TEM hydrogels can markedly enhance the bioactivity and function of thyroid cells in an *in vitro* 3D culture system. This provides crucial experimental evidence for thyroid tissue engineering and regenerative medicine research. Although the current study demonstrates the significant advantages of high-concentration TEM hydrogel in promoting thyroid cell viability and function, further research is needed to explore its potential and mechanisms *in vivo*. Future studies could include animal experiments to validate the effectiveness of TEM hydrogels in thyroid tissue repair and regeneration, as well as investigate their safety and efficacy in clinical applications. Additionally, further research on the specific components of the TEM and their roles in regulating cell function could help optimize the preparation process and application efficacy of TEM hydrogels, providing a more robust theoretical and experimental basis for thyroid regenerative medicine research.

## Data Availability

The raw data supporting the conclusions of this article will be made available by the authors, without undue reservation.
